# Establishment of a prognostic nomogram for patients with locoregionally advanced nasopharyngeal carcinoma incorporating clinical characteristics and dynamic changes in hematological and inflammatory markers

**DOI:** 10.3389/fonc.2022.1032213

**Published:** 2022-10-26

**Authors:** Qin Liu, Lingyu Ma, Huangrong Ma, Li Yang, Zhiyuan Xu

**Affiliations:** ^1^ Clinical Oncology Center, The University of Hong Kong-Shenzhen Hospital, Shenzhen, Guangdong Province, China; ^2^ Shenzhen Key Laboratory of Translational Research on Recurrent/Metastatic Cancer, The University of Hong Kong - Shenzhen Hospital, Shenzhen, Guangdong Province, China

**Keywords:** nasopharyngeal carcinoma, nomogram, immune cells, prognosis, chemo-radiation

## Abstract

**Background:**

This study aims to investigate the prognostic value of changes in hematological and inflammatory markers during induction chemotherapy (IC) and concurrent chemo-radiation (CCRT), thus construct nomograms to predict progression free survival (PFS) of patients with locally advanced nasopharyngeal carcinoma (LANPC).

**Methods:**

130 patients were included in this prospective analysis. Univariate and multivariate cox regression analyses were conducted to identify prognostic factors. Three multivariate analyses integrating different groups of variables were conducted independently. Concordance indexes (c-index), calibration plots and Kaplan-Meier curves were used to evaluate the nomograms. Bootstrap validation was performed to determine the accuracy of the nomogram using 1000 resamples. The performances of proposed nomograms and TNM staging system were compared to validate the prognostic value of hematological and inflammatory markers.

**Results:**

Pretreatment gross tumor volume of nodal disease (GTVn), Δe/bHGB (hemoglobin count at end of treatment/baseline hemoglobin count), and stage were selected as predictors for 3-year PFS in first multivariate analysis of clinical factors. The second multivariate analysis of clinical factors and all hematological variables demonstrated that ΔminLYM (minimum lymphocyte count during CCRT/lymphocyte count post-IC), pretreatment GTVn and stage were associated with 3-year PFS. Final multivariate analysis, incorporating all clinical factors, hematological variables and inflammatory markers, identified the following prognostic factors: pretreatment GTVn, stage, ΔmaxPLR (maximum platelet-to-lymphocyte ratio (PLR) during CCRT/PLR post-IC), and ΔminPLT (minimum platelet count during CCRT/platelet count post-IC). Calibration plots showed agreement between the PFS predicted by the nomograms and actual PFS. Kaplan–Meier curves demonstrated that patients in the high-risk group had shorter PFS than those in the low-risk group (*P ≤* 0.001). The c-indexes of the three nomograms for PFS were 0.742 (95% CI, 0.639-0.846), 0.766 (95% CI, 0.661-0.871) and 0.815 (95% CI,0.737-0.893) respectively, while c-index of current TNM staging system was 0.633 (95% CI, 0.531-0.736).

**Conclusion:**

We developed and validated a nomogram for predicting PFS in patients with LANPC who received induction chemotherapy and concurrent chemo-radiation. Our study confirmed the prognostic value of dynamic changes in hematological and inflammatory markers. The proposed nomogram outperformed the current TNM staging system in predicting PFS, facilitating risk stratification and guiding individualized treatment plans.

## Introduction

Nasopharyngeal carcinoma (NPC) demonstrates high prevalence in southern China, Southeast Asia, and North Africa (1). It was estimated that there were 133,354 new cases worldwide, with 80,008 deaths in 2020 (2). Despite substantial improvement in radiotherapy techniques and concurrent chemotherapy for NPC, many patients suffer from local-regional recurrence and distant metastasis (3). A meta-analysis by Chiang et al. identified a set of significant prognostic factors including primary gross tumor volume (GTV), Epstein-Barr virus (EBV)-DNA level, lactate dehydrogenase (LDH), C-reactive protein/albumin ratio, platelet count, and the maximum standard uptake value (SUVmax) of the primary tumor (4). In particular, there is a strong association between NPC and EBV infection. Heavy infiltration of immune cells surrounding and within the tumor is one of the pathological hallmarks of NPC, indicating a complex tumor microenvironment (TME) and the potential benefits of immunotherapy (1, 5–7). Therefore, several other prognostic factors reflective of the biological behavior of tumors have been identified to predict the survival outcome of patients with NPC. These include hematological and immunological markers such as hemoglobin, lymphocytes, neutrophil-to-lymphocyte ratio (NLR), and platelet-to-lymphocyte ratio (PLR) (8–15). There are growing interests in incorporating these factors/biomarkers into the current TNM staging system to further improve risk stratification. Many groups have attempted to develop nomograms to guide the stratified treatment of patients with NPC (16–19). However, prognostic values of lymphocytes and other immune cells were not always assessed in the analyses, or only pretreatment levels were included. Indeed, lymphocyte counts and other hematological or inflammatory variables do not remain static during treatment. Radiation-induced lymphopenia is associated with survival in many cancer types (20–28). Recovery of severe lymphopenia after treatment has been associated with better survival in pancreatic cancer (29). In patients with NPC, the midradiation hemoglobin level was an important predictive factor for local control and survival. The high incidence of anemia following chemotherapy has a negative predictive value for treatment outcomes and may diminish the efficacy of induction chemotherapy (15). Hemoglobin levels before and during radiotherapy appear significant for treatment outcome (30). This evidence indicates the potential predictive role of dynamic changes in blood cells. Therefore, we intend to comprehensively investigate the predictive value of hematological and inflammatory markers, including both their pre-treatment baseline and treatment-induced changes.

Thus, we aim to develop a more comprehensive prognostic nomogram integrating clinical factors and dynamic changes in hematological and inflammatory markers during induction chemotherapy (IC) and concurrent chemo-radiation (CCRT) in locally advanced NPC.

## Methods

### Patients

Between January 2015 to September 2019, a total of 130 patients who attended the University of Hong Kong-Shenzhen Hospital were enrolled in this prospective analysis. Patients included were newly diagnosed biopsy-proven NPC with stage III to IVB disease as defined by the 7th edition of the American Joint Committee on Cancer–Union for International Cancer Control (AJCC-UICC TNM-7) before 2018 or stage III-IVA disease based on AJCC-UICC TNM-8 since 2018 (except T3N0). Patients recruited before 2018 were restaged using AJCC-UICC TNM-8 by two independent oncologists prior to the analysis of this research. Any discrepancy in staging was resolved by consensus. Patients with a prior history of malignancy or anti-cancer treatment or those not eligible for radical treatment were excluded. This study was approved by the Clinical Research Ethics Committee of the University of Hong Kong -Shenzhen Hospital. Informed consents were obtained from all participants.

### Data collection

The following baseline information was collected prior to the start of treatment: sex, age, TNM staging, histology, full blood count (FBC), renal function test (RFT), liver function test (LFT), thyroid function, morning cortisol and plasma EBV DNA. History including family history, smoking and alcohol history, and comorbidity assessment are part of the diagnostic workup. The ECOG performance status, nutritional status, primary and nodal GTV volume before and after induction chemotherapy and RT dosimetry were also collected. Blood tests, including FBC, RFT and LFT, were repeated before each cycle of chemotherapy as well as before and after radiotherapy.

The calculation formulas of inflammatory markers are as follows:

PLR (platelet-to-lymphocyte ratio) = platelet count (10^9^/L)/lymphocyte count (10^9^/L);
NLR (neutrophil-to-lymphocyte ratio) = neutrophil count (10^9^/L)/lymphocyte count (10^9^/L);
SII (systemic inflammatory index) = platelet count (10^9^/L) x neutrophil count (10^9^/L)/lymphocyte count (10^9^/L).

Dynamic changes of hematological and inflammatory makers are presented as follows:

ΔminLYM = minimum lymphocyte count during CCRT/lymphocyte count post-IC;
Δp/bLYM = lymphocyte count post-IC/baseline lymphocyte count;
Δe/bLYM = lymphocyte count at end of treatment/baseline lymphocyte count;
Δe/pLYM = lymphocyte count at end of treatment/lymphocyte post-IC;
ΔmaxNLR = maximum NLR during CCRT/NLR post-IC.

Similar methods were applied to calculate changes in hemoglobin (HGB), neutrophils (NEUT), platelet (PLT), calcium level (CA), NLR, PLR and SII (**Supplementary Table 1**).

### Treatment and follow-up

All patients were treated with induction chemotherapy followed by concurrent chemo-radiation. The induction PX regime consisted of cisplatin at 80 mg/m2 on day 1 every 3 weeks and oral capecitabine at 1000 mg/m2 twice daily from day 1 to 14, for 3 cycles. Concurrent cisplatin was administered intravenously at a dose of 100 mg/m2 on day 1 every 3 weeks for 2 cycles. Dose reduction was allowed as clinically indicated. Our institute followed the International Consensus Guidelines for Target delineation and OAR dose constraints (31, 32). All patients were treated with radical RT using intensity-modulated radiotherapy (IMRT) or volumetric modulated arc therapy (VMAT) techniques. Total doses of 70 Gy, 63 Gy, and 56 Gy in 35 fractions over 7 weeks were prescribed to high, intermediate, and low risk planning target volumes (PTV), respectively. All patients were followed up every three months in the first three years and biannually until death.

### Statistical analysis

Progression-free survival (PFS) was defined as the time from the start of treatment to tumor progression or death from any cause or the date of the last follow-up. Continuous variables were shown as medians with interquartile ranges (IQRs), whereas categorical variables were presented as numbers and proportions. The kernel-weighted polynomial smoothing method was used to demonstrate blood count kinetics using all available FBCs. The time effects were analyzed with generalized estimating equation (GEE) using the Stata xtgee procedure. The multivariate Cox regression was performed on variables with a *P* value < 0.1 in the univariate analysis.

The nomograms were constructed based on the results of the multivariate analysis by backward stepwise selection with the Akaike Information Criterion (AIC). The proportional hypotheses of the final models were validated. Multicollinearity was evaluated by the variance inflation factor (VIF) and spearman rank correlation.

Variables Selection and Model Development based on different inclusions of variables, three models were developed to predict the PFS in locally advanced NPC patients treated with induction chemotherapy and CCRT: (a) candidate predictors for Nomogram 1 were limited to the clinical variables and hemoglobin only; (b) candidate predictors for Nomogram 2 added other parameters in FBCs that are lymphocyte, neutrophil and platelet counts; (c) candidate predictors for Nomogram 3 included all variables listed in **Table 1**. Finally, the dose-response association between variables and PFS was examined using Restricted Cubic Splines (RCS) with three knots at the 10th, 50th, and 90th percentiles.

**Table 1:** Patient characteristics and univariate analysis.

Variables	Group	Median (IQR), n(%)	Progression free survival	
			HR (95%CI)	*P*-value
Clinical factors				
Age	Continuous	44 (37, 53)	1.023 (0.985, 1.062)	0.234
Sex	Female	39 (30)	Reference	
	Male	91 (70)	1.386 (0.508, 3.784)	0.524
RT Techniques	VMRT	95 (73.08)	Reference	
	IMRT	35 (26.92)	1.142 (0.454, 2.875)	0.777
ECOG score	0	9 (6.92)	Reference	
	≥1	121 (93.08)	0.478(0.140, 1.630)	0.238
N classification^*^	N0-1	18 (13.84)	Reference	
	N2	80 (61.54)	0.427 (0.125, 1.465)	0.176
	N3	32 (24.62)	1.695 (0.528, 5.435)	0.375
T classification^*^	T1-2	40 (30.77)	Reference	
	T3	63 (48.46)	0.447 (0.155, 1.288)	0.136
	T4	27 (20.77)	1.229 (0.445, 3.392)	0.691
Clinical stage^*^	III	76 (58.46)	Reference	
	IV	54 (41.54)	3.008 (1.214, 7.454)	0.017
BSA (m2)	Continuous	1.714 (1.582, 1.823)	1.116 (0.095, 13.060)	0.930
Baseline EBV-DNA (copies/ml)	<5000	119 (91.54)	Reference	
	(≥5000)	10 (7.69)	3.377 (1.133, 10.062)	0.029
Mean body dose (Gy)	Continuous	14.623 (13.355, 18.200)	1.046 (0.935, 1.169)	0.434
Total dose (Gy)	Continuous	17.202 (15.870, 21.475)	1.055 (0.936, 1.189)	0.380
Histology	WHO type 2	15 (11.54)	Reference	
	WHO type 3	108 (83.08)	1.133 (0.263, 4.888)	0.867
	Undetermined	7 (5.38)	0.904 (0.082, 9.972)	0.934
Baseline BMI	Continuous	23.300 (20.800, 25.325)	0.975 (0.849, 1.121)	0.725
End of treatment BMI	Continuous	23.172 (20.862, 25.238)	0.997 (0.870, 1.143)	0.971
ΔBMI	Continuous	1 (0.977, 1.014)	32.847 (0,13849591.987)	0.597
Smoking	Yes	24 (18.46)	Reference	
	No	106 (81.54)	0.659 (0.241, 1.803)	0.417
Drinking	Yes	12 (9.23)	Reference	
	No	118 (90.77)	0.748 (0.173, 3.232)	0.697
Family history	Yes	8 (6.15)	Reference	
	No	122 (93.85)	1.298 (0.174, 9.677)	0.799
Complications	Yes	20 (15.38)	Reference	
	No	110 (84.62)	1.116 (0.328, 3.792)	0.861
Induction chemotherapy	Standard dose	123 (94.62)	Reference	
	Reduced dose	7 (5.38)	2.121 (0.494, 9.114)	0.312
Concurrent chemotherapy	Standard dose	96 (73.85)	Reference	
	Reduced dose	34 (26.15)	1.617 (0.650, 4.020)	0.301
Δp/bCA	Continuous	0.991 (0.958, 1.016)	260004.838 (13.44,5029782353.027)	0.013
Grade 3-4 AE during IC^#^	No	102 (78.46)	Reference	
	Yes	28 (21.54)	0.863 (0.290, 2.568)	0.791
Grade 3-4 AE^#^	No	28 (21.54)	Reference	
	Yes	102 (78.46)	1.745 (0.514, 5.926)	0.372
GTVp_preIC (cm3)(log)	Continuous	1.495 (1.313, 1.764)	1.352 (0.297, 6.163)	0.697
GTVp_postIC (cm3)(log)	Continuous	1.175 (0.889, 1.424)	1.987 (0.573, 6.892)	0.279
GTVn_preIC (cm3)(log)	Continuous	1.378 (1.139, 1.64)	6.481 (2.207, 19.034)	0.001
GTVn_postIC (cm3)(log)	Continuous	0.832 (0.580, 1.147)	4.438 (1.721, 11.445)	0.002
Hematological variables				
Δp/bLYM	Continuous	0.913 (0.754, 1.107)	0.485 (0.091, 2.588)	0.397
Δe/bLYM	Continuous	0.207 (0.171, 0.316)	2.651 (0.396, 17.744)	0.315
Δe/pLYM	Continuous	0.236 (0.171, 0.375)	7.214 (1.114, 46.701)	0.038
ΔminLYM	Continuous	0.250 (0.190, 0.330)	158.872 (3.070, 8222.559)	0.012
Δp/bPLT	Continuous	0.680 (0.569, 0.805)	0.384 (0.042, 3.538)	0.398
Δe/bPLT	Continuous	0.812 (0.623, 0.971)	0.844 (0.257, 2.774)	0.780
Δe/pPLT	Continuous	1.128 (0.887, 1.432)	1.236 (0.474, 3.220)	0.665
ΔminPLT	Continuous	0.611 (0.463, 0.782)	10.400 (1.283, 84.339)	0.028
Δp/bNEUT	Continuous	0.533 (0.349, 0.737)	0.964 (0.631, 1.474)	0.867
Δe/bNEUT	Continuous	0.695 (0.476, 1.100)	0.861 (0.465, 1.596)	0.635
Δe/pNEUT	Continuous	1.311 (0.839, 2.127)	0.921 (0.717, 1.183)	0.519
ΔminNEUT	Continuous	0.657 (0.303, 0.888)	1.096 (0.271, 4.431)	0.897
Δp/bHGB	Continuous	0.795 (0.730, 0.864)	21.191 (0.271,1658.660)	0.170
Δe/bHGB	Continuous	0.683 (0.619, 0.741)	19.878 (1.106,357.273)	0.043
Δe/pHGB	Continuous	0.864 (0.768, 0.963)	4.304 (0.192, 96.260)	0.357
ΔminHGB	Continuous	0.799 (0.731, 0.870)	3.582 (0.045, 282.150)	0.567
Inflammatory markers				
Δp/bNLR	Continuous	0.613 (0.374, 0.897)	1.025 (0.628, 1.673)	0.922
Δe/bNLR	Continuous	3.159 (1.978, 5.278)	0.868 (0.712, 1.059)	0.163
Δe/pNLR	Continuous	4.827 (2.925, 8.929)	0.924 (0.828, 1.031)	0.158
ΔmaxNLR	Continuous	9.851 (5.996, 15.514)	0.959 (0.905, 1.016)	0.156
Δp/bSII	Continuous	0.425 (0.240, 0.684)	0.972 (0.517, 1.829)	0.931
Δe/bSII	Continuous	2.547 (1.229, 4.853)	0.882 (0.736, 1.058)	0.176
Δe/pSII	Continuous	5.971 (2.698, 11.630)	0.949 (0.878, 1.026)	0.190
ΔmaxSII	Continuous	10.203 (6.244, 17.099)	0.956 (0.902, 1.014)	0.135
Δp/bPLR	Continuous	0.765 (0.578, 0.957)	0.739 (0.185, 2.942)	0.667
Δe/bPLR	Continuous	3.529 (2.193, 4.773)	0.866 (0.706, 1.061)	0.164
Δe/pPLR	Continuous	4.585 (2.635, 7.085)	0.908 (0.780, 1.058)	0.217
ΔmaxPLR	Continuous	6.589 (4.730, 8.918)	0.859 (0.731, 1.010)	0.066

IQR, interquartile range; HR, hazard ratio; CI, confidence interval; IMRT, intensity-modulated radiotherapy; VMAT, volumetric modulated arc therapy; ECOG, Eastern Cooperative Oncology Group; BSA, body surface area; BMI, body mass index; CA, calcium; AE, adverse events; IC, induction chemotherapy; GTVp, gross tumor volume of primary tumor; GTVn, gross tumor volume of lymph nodes; BMI, body mass index; GTVp_preIC, GTVp volume before induction chemotherapy; GTVp_postIC, GTVp volume after induction chemotherapy; GTVn_preIC, GTVn volume before induction chemotherapy; GTVn_postIC, GTVn volume after induction chemotherapy; ^*^Staged according to the 8th edition of the American Joint Committee on Cancer–Union for International Cancer Control tumor-node-metastasis (TNM) cancer staging system (AJCC-UICC TNM-8); ^#^AE was graded by the Common Terminology Criteria for Adverse Events version 4.0 (CTCEA v4.0).

The performance of each nomogram to differentiate patients with different outcomes was assessed by discriminating ability and calibration. The concordance index (c-index) was used to evaluate the prediction accuracy of the nomogram. The rcorrp.cens function was used to generate *P* values between C-statistics. Bootstrap verification with 1000 resamples was used to quantify the prediction accuracy of the nomogram. Patients were divided into two risk groups according to risk scores calculated using nomogram. The optimal cut-off values for dividing low-risk and high-risk groups were identified using X-tile software (Rimm Laboratory, Yale School of Medicine, New Haven, CT, USA). The Kaplan-Meier method was employed. The Log-rank test was performed to assess the differences between survival curves. GTVs were log-transformed prior to analysis. R version 3.6.2 (http://www.r-project.org), Stata version 12 (StataCorp, College Station, TX, USA), and SPSS version 25 (IBM, Armonk, NY, USA) were used for all statistical analysis procedures. All *P* values were bilateral, and statistical significance was defined as *P* < 0.05. Sensitivity analysis was performed to prevent potential selection bias by discarding patients with missing predictor information. 10 completed datasets were imputed, and the univariable and multivariable procedure was repeated in each completed dataset. Variables for the final model were selected if they were retained in >5 datasets. The pooled estimates were used to account for statistical uncertainty inherent to the imputation.

## Results

### Patient characteristics

A total of 130 cases were enrolled in this study. Patient characteristics are shown in **Table 1**. 58.46% of patients were diagnosed with stage III NPC and the rest 41.54% were diagnosed with stage IVA disease. 94.62% of patients completed induction chemotherapy with the standard dose. Only 7 patients required dose reduction. 21.54% of patients experienced grade 3-4 adverse events during induction chemotherapy, while during concurrent chemotherapy, 78.46% experienced grade 3-4 adverse events and 26.15% of patients required dose reduction. The median follow-up was 52.5 (48.4-56.5) months. The 3-years PFS rate was 86% (95% CI, 80%-92%).

**Figure 1:**
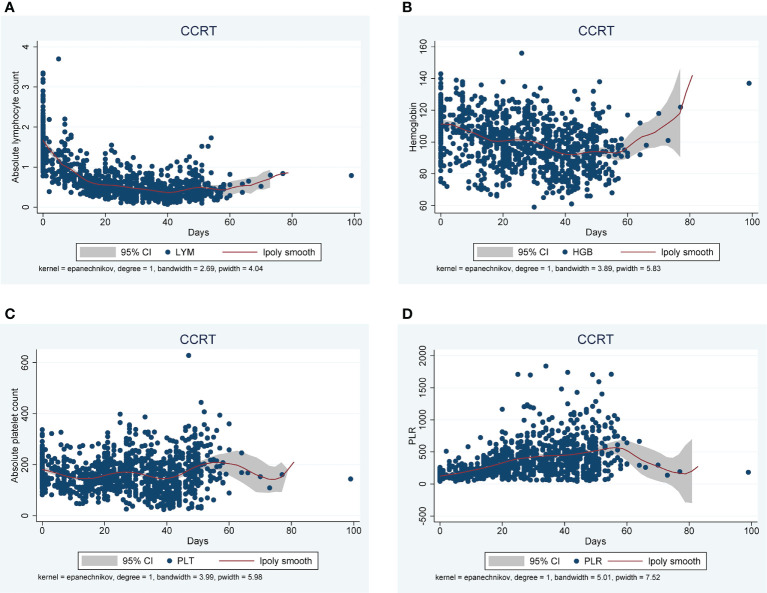
Changes in hematological parameters during CCRT, including absolute lymphocyte count (LYM)(10^9^/L) **(A)**, hemoglobin(HGB) (g/L) **(B)**, absolute platelet count (PLT)(10^9^/L) **(C)**, and platelet-to-lymphocyte ratio (PLR)**(D)**.

Analysis of FBC parameters revealed that IC has minimal effect on absolute lymphocyte counts (**Supplementary Figure 1B** and **Table 2**). Nevertheless, lymphocyte count reduced significantly during CCRT (**Figure 1A** and **Supplementary Table 2**). Grade 3 lymphopenia appeared as early as week 1 and grade 4 lymphopenia was observed at week 4 of CCRT (**Supplementary Figure 3**). In contrast, neutrophil counts did not change significantly during treatment, probably due to the use of granulocyte colony-stimulating factor (G-CSF) (**Supplementary Figures 1C, 2A** and **Supplementary Table 4**). Both hemoglobin and absolute platelet counts decreased significantly during treatment (**Figures 1B, C**; **Supplementary Figure 1A, D** and **Supplementary Table 3, 5**). Inflammatory markers, such as NLR, PLR and SII, increased significantly during treatment (**Figure 1D**; **Supplementary Figures 1E–G, 2B, C**, and **Supplementary Tables 6–8**).

**Table 2:** Multivariate Cox analysis.

Variables	Nomogram 1		Nomogram 2		Nomogram 3	
	HR (95%CI)	*P*-value	HR (95%CI)	*P*-value	HR (95%CI)	*P*-value
Log GTVn-preIC	4.673 (1.630, 13.402)	0.004	4.697 (1.631, 13.528)	0.004	3.976 (1.382, 11.440)	0.010
Clinical stage	2.197 (0.855, 5.645)	0.102	2.764 (1.071, 7.134)	0.036	2.792 (1.078, 7.228)	0.034
Δe/bHGB	14.837 (0.997, 220.807)	0.050	–	–	–	–
ΔminLYM	–	–	284.066 (3.991, 20220.747)	0.009	–	–
ΔminPLT	–	–	–	–	5.914 (0.561, 62.330)	0.139
ΔmaxPLR	–	–	–	–	0.819 (0.689, 0.974)	0.024

HR, hazard ratio; CI, confidence interval; GTVn_preIC, GTVn volume before induction chemotherapy.

### Development of nomograms

Univariate analysis showed that stage, baseline EBV-DNA level, nodal GTV volume, treatment-induced changes in hemoglobin level (Δe/bHGB), platelet counts (ΔminPLT), lymphocytes (Δe/pLYM and ΔminLYM) and calcium (Δp/bCA) were associated with PFS (**Table 1**).

**Figure 2:**
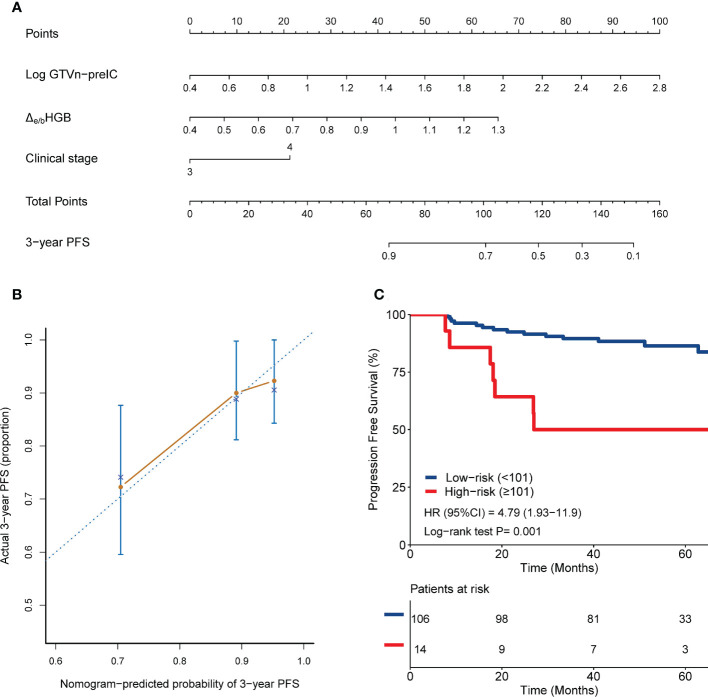
Nomogram 1 integrating clinical stage, pretreatment GTVn and Δe/bHGB for predicting 3-year PFS **(A)**; calibration plots of survival probabilities at 3-year **(B)**; Kaplan-Meier survival curves for patients stratified by nomogram 1 **(C)**.

Three multivariate analyses were conducted independently. First multivariate analyse identified that Δe/bHGB (HR,14.837; 95% CI, 0.997-220.807; *P*=0.05), pretreatment GTVn volume (HR, 4.673; 95% CI, 1.630-13.402; *P*=0.004) and stage (HR, 2.197; 95% CI, 0.855-5.645; *P*=0.102) were associated with PFS (**Table 2**). First nomogram was constructed based on these prognostic factors (**Figure 2A**).

**Figure 3:**
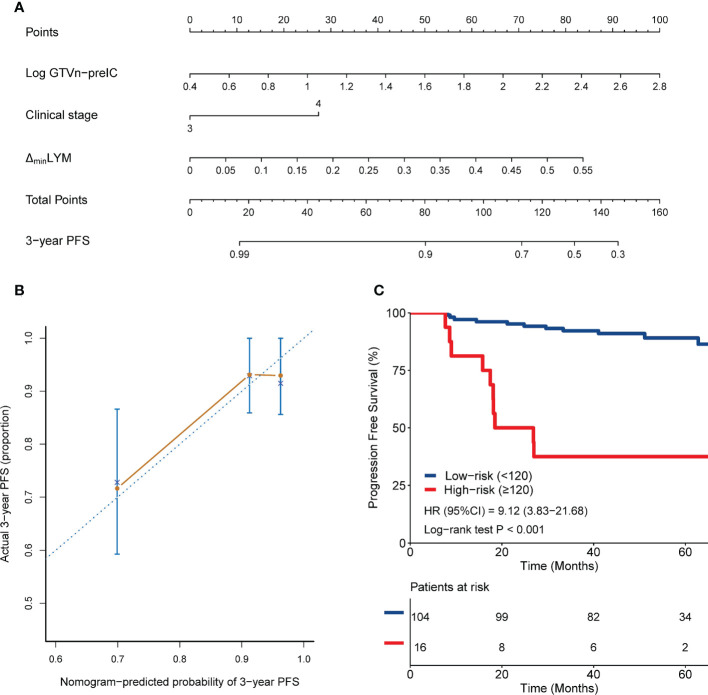
Nomogram 2 integrating clinical stage, pretreatment GTVn and ΔminLYM for predicting 3-year PFS **(A)**; calibration plots of survival probabilities at 3-year **(B)**; Kaplan-Meier survival curves for patients stratified by nomogram 2 **(C)**.

Second multivariate analysis added hematological variables to clinical factors (**Table 1**) and demonstrated ΔminLYM (HR, 284.066; 95% CI, 3.991-20220.747; *P*=0.009), pretreatment GTVn (HR;4.697; 95% CI, 1.631-13.528; *P*=0.004) and stage (HR, 2.764; 95% CI, 1.071-7.134, *P*=0.036) were independently associated with 3-year PFS (**Table 2** and **Figure 3A**).

**Figure 4:**
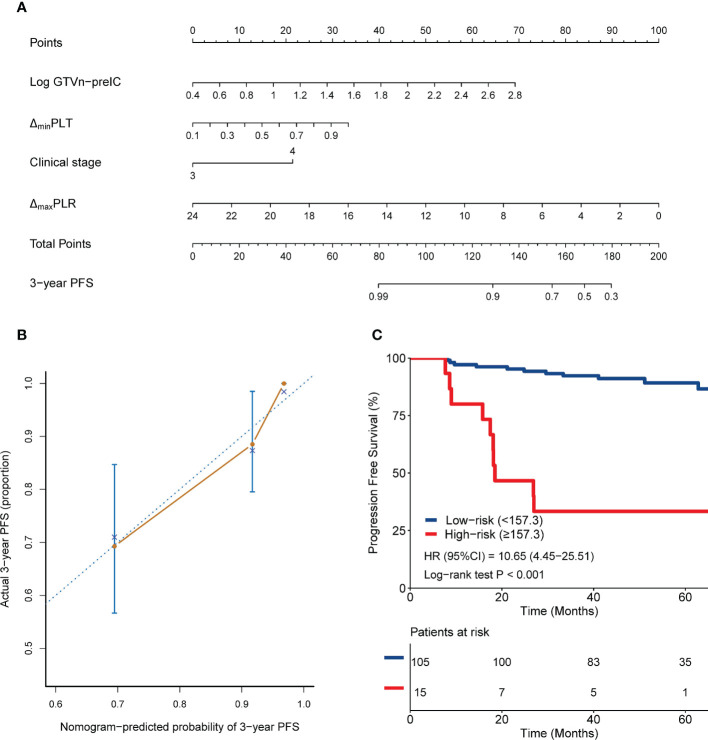
Nomogram 3 integrating clinical stage, pretreatment GTVn, ΔmaxPLR and ΔminPLT for predicting 3-year PFS **(A)**; calibration plots of survival probabilities at 3-year **(B)**; Kaplan-Meier survival curves for patients stratified by nomogram 3 **(C)**.

Third nomogram was built on the basis of the third multivariate analysis (**Figure 4A**), which identified the following prognostic factors amongst all clinical factors, hematological variables and inflammatory markers with *P* < 0.1 in the univariate analyses: pretreatment GTVn volume (HR,3.976; 95% CI, 1.382-11.440; *P*=0.01), stage (HR, 2.792; 95% CI, 1.078-7.228; *P*=0.034), ΔmaxPLR (HR, 0.819; 95% CI, 0.689-0.974; *P*=0.024) and ΔminPLT (HR, 5.914; 95% CI, 0.561-62.330; *P*=0.139) (**Table 2**). The total points can be used to determine the survival probability by aligning it with the total scale.

### Validation of the nomograms for PFS and in comparison with TNM staging

Calibration plots demonstrated agreement between the nomogram predicted PFS and actual PFS (**Figures 2B**, **3B**, **4B**). The c-index of nomogram 1, 2 and 3 for PFS were 0.742 (95% CI, 0.639-0.846), 0.766 (95% CI, 0.661-0.871) and 0.815 (95%CI, 0.737-0.893) respectively (**Table 3**). The model incorporating hematological and inflammatory markers (nomogram 3) demonstrated the best discriminative ability. The c-index of the TNM staging system was 0.633 (95%CI, 0.531-0.736), and the AIC value of the TNM staging system for PFS was 192.6309. Compared with nomogram 3, the TNM staging system yielded a significantly lower c-index (P < 0.001). Bootstrap validation was performed to determine the accuracy of the nomogram using 1000 resamples (**Table 3**).

**Table 3:** Performances of current TNM staging system and proposed nomograms.

	AIC	c-index	95%CI	c-index (bootstrap)	*P*-value
TNM Stage	192.631	0.633	0.531-0.736	0.631	Reference
Nomogram 1	181.755	0.742	0.639-0.846	0.715	0.027
Nomogram 2	176.965	0.766	0.661-0.871	0.744	0.010
Nomogram 3	175.159	0.815	0.737-0.893	0.761	<0.001

TNM, tumor–node–metastasis; AIC, Akaike Information Criterion; c-index, concordance index.

### Performance of the nomograms in risk stratification

Kaplan–Meier curves were plotted for each nomogram to assess the discriminative power of the nomograms (**Figures 2C**, **3C**, **4C**). Patients were stratified into high-risk and low-risk groups based on cut-off values of 101, 120, and 157.3 points, respectively. Patients in the high-risk groups had significantly worse PFS than those in the low-risk groups (*P≤* 0.001).

## Discussion

In this study, we constructed three nomograms predicting the PFS for patients with locally advanced NPC treated with induction chemotherapy and concurrent chemo-radiation. The first nomogram included clinical variables and hemoglobin only. The second nomogram added absolute lymphocyte, neutrophil and platelet counts. The third nomogram included all factors in the first two nomograms as well as PLR, NLR and SII. Improvement in c-indexes can be seen with the addition of hematological parameters and inflammatory markers, suggesting the importance of these biomarkers in predicting patient outcomes. The final nomogram composed of pretreatment GTVn, TNM stage, ΔmaxPLR and ΔminPLT produced a robust model to predict survival outcome with a c-index of 0.815.

The AJCC-UICC TNM-8 is the most utilized tool to predict patient outcomes and guide treatment planning and remains the most robust tool for global application. However, there are limitations to the current TNM staging system. Tang et al. reported that the eighth edition classification system had unsatisfactory results in separating the survival of curves between T2 and T3 disease (33). Jen et al. also supported the limitation of the current staging system (34). In recent years, there has been growing interest in incorporating non-anatomical prognostic factors into the staging system. These variables may be relevant for individualized risk stratification and guiding the treatment intensity (4).

Across three nomograms, pretreatment GTVn volume and TNM stage were consistently identified as important prognostic factors. In the second nomogram, treatment-induced change in lymphocyte counts, described as ΔminLYM, outperformed hemoglobin as a better prognostic factor. Lymphocyte counts remained relatively stable during and after induction chemotherapy, and decreased significantly during CCRT. This change has been associated with incidental bone marrow irradiation (35). Radiation can negatively impact multipotent mesenchymal stem cells at an unexpectedly low dose (36). The mechanism of apoptosis in lymphocytes was associated with the up-regulation of CD95/Fas/APO-1 ligand (37). Tumor cells expressing Fas ligand (FasL) are involved in counterattacks to eliminate tumor-infiltrating lymphocytes. Increased expression of FasL facilitates tumor development and metastasis by escaping immune surveillance (38). Apart from FasL, cytokines such as IL-10 and IL-6, have been demonstrated to play important roles in immunosuppression and promote tumor growth (39–41). Serum levels of IL-6 were increased following radiation or chemo-radiation therapy in head and neck cancer (40). The IL-6/JAK/STAT3 signaling pathway is hyperactivated in many cancer types and leads to a strong suppression of the anti-tumor immune response (42). Radiation-induced FasL overexpression and the rise of pro-inflammatory cytokines leading to lymphopenia may promote cancer growth (35). Nevertheless, the role of cytokines has yet to be thoroughly studied, particularly their interplay with lymphocyte counts.

When non-linear relationships between lymphocyte counts and PFS were modeled using RCS, we found that a ΔminLYM ≥0.158 was associated with worse PFS (**Supplementary Figure 4**). Peripheral blood lymphocytes are crucial in mediating cellular immunity against neoplastic cells. Lymphopenia and its prognostic value have been studied in various cancer types. Studies showed that lymphopenia is associated with worse outcomes in esophageal cancer (26), high-grade glioma (21), pancreatic cancer (25), cervical cancer (28), and non-small cell lung cancer (21). Interestingly lymphopenia does not always relate to a poor prognosis. Treatment-related lymphopenia was seen following pelvis nodal irradiation in patients with prostate cancer. However, it was not predictive of biochemical progression-free survival, distant metastasis, or overall survival (43). In two studies on anal cancer, controversial results were found. Lee et al. reported a 3.7-fold increase in death in patients with treatment-related lymphopenia (22). However, a more recent study with a larger cohort showed that lymphopenia during and after chemo-radiation for anal cancer was not associated with worse survival, recurrence, or metastases (20). This difference in the conclusions may be owing to the small sample size and lower baseline lymphocyte counts in Lee’s study. Therefore, the nominal difference in OS may be driven by small sample size and lower baseline could potentially confound the interpretation of the results (20). Similarly, a study with a large cohort of oropharyngeal cancer patients demonstrated no association between treatment-related lymphopenia and clinical outcomes (24). Although we see increasing data published in solid tumors, the association between radiation-induced lymphopenia (RIL) and adverse oncologic outcomes remains inconclusive. Therefore, further data are eagerly sought to clarify the actual effect of treatment-induced lymphopenia on survival.

In head and neck cancers, 60% of patients treated with chemo-radiation had treatment-related lymphopenia. Severe lymphopenia in human papillomavirus negative (HPV-) patients is independently associated with earlier disease progression (44). With regards to NPC, we found three previous studies on lymphopenia and its effect on patient outcomes with contradictory conclusions. Two studies reported lymphopenia as a poor prognostic factor for survival (12, 14). Specifically, Liu et al. reported that a minimum absolute lymphocyte count (ALC) < 0.39× 10^9^ cells/L indicates an early 2-fold increase in the risk of early death (12). Contrary to these findings, one recent study by Xie et al. showed that lymphopenia was associated with better outcomes. This retrospective study, including 374 patients with stage II-IVa treated with definitive RT, reported that grade 3-4 lymphopenia (ALC< 0.5 × 10^9^ cells/L) was independently associated with longer PFS and LRFS. In contrast, grade 4 lymphopenia (ALC < 0.2 × 10^9^ cells/L) was associated with a shorter DMFS (13). It is worth noting that Liu’s and Xie’s study used different cut-offs for lymphopenia and RT techniques. Given that grade 4 lymphopenia was associated with shorter DMFS while grade 3-4 combined was not associated with a change in DMFS, it is possible that results from Liu’s study may be skewed by patients with ALC < 0.2 × 10^9^ cells/L. Nevertheless, the major difference in conclusions from these two studies was the prognostic value of lymphopenia in PFS. Xie et al. reported longer PFS in patients with grade 3-4 lymphopenia, while Liu et al. reported shorted PFS in patients with treatment-related lymphopenia. The present study showed that lymphopenia is associated with longer PFS in patients with NPC receiving concurrent chemo-radiation. The reason why treatment-related lymphopenia is related to favorable survival outcomes in NPC may be difficult to elucidate. Here, we discuss several possible explanations.

Firstly, lymphocytes include T cells, B cells and natural killer (NK) cells. T cells can further divide into CD4+ T lymphocytes and CD8+ T lymphocytes. A recent meta-analysis elucidated the changes in T lymphocyte subtypes before and after RT (45). Overall, CD4+ lymphocytes reduced significantly after RT, while CD8+ lymphocytes did not change significantly. This means lymphocyte subtypes may respond differently to radiation. CD8+ T lymphocytes, memory T lymphocytes, and regulatory T cells are less sensitive to radiation than B cells, CD4+ T cells, and perhaps naive T cells (45). Furthermore, different lymphocyte subtypes respond differently to different treatment modalities. CD3+ lymphocytes decreased significantly after RT alone, while there was no significant change after CCRT. CD4+ lymphocytes, on the other hand, reduced significantly in number following CCRT and remained stable after RT, while the number of CD8+ lymphocytes increased with CCRT (45). As lymphocytes also plays an immune suppressive role, further studies on lymphocyte subtypes are needed to clarify the underlying mechanism of lymphocytes and their antitumor effect.

Secondly, the distribution of lymphocyte subtypes is heterogenous (46). The distribution of circulating lymphocytes can be quite different after RT even in patients who have received radiation at the same site. In addition, cancer progression results in lymphocyte infiltration into tumors and migration to tumor-draining lymph nodes (14, 47). Hence, assessing tumor-infiltrating lymphocytes using tissue biopsies at consecutive time points might yield more valuable results, although such an approach would be challenging to implement in clinical settings. Notably, Wang et al.’s study revealed that T cells were reduced following radiotherapy for head and neck cancer, while such decrease was not observed in prostate and breast cancer. Moreover, RT may have proliferative and activating effects on T cells in esophageal and lung tumors (45). These findings demonstrated the uniqueness of head and neck cancer and offered a possible explanation for why our findings in NPC differ from those in other cancer types.

Lastly, the hypothesis of crosstalk between primary tumor and metastasis has been supported by experimental and clinical evidence. In T cell-deficient (nude) mice with implanted human pancreatic carcinoma at two separate sites, irradiation of one tumor with concomitant capecitabine resulted in marked inhibition of non-irradiated tumor. As the mice lacked T cells, the antitumor effect may be contributed by other cytokines. It leads to the controversy if T cells are required for abscopal effects, cytokines may also induce tumor cell death, and innate immune response and dendritic cells play an important role in host antitumor response (47). This observation may explain why a lack of lymphocytes does not necessarily mean a poor prognosis.

In the third nomogram, ΔminPLT and ΔmaxPLR combined outperformed lymphocyte counts as prognostic factors for PFS. Higher ΔminPLT during CCRT is found to be associated with poorer prognosis. To our best knowledge, we are the first to study the prognostic effects of platelet levels before, during and after IC and CCRT. Several studies have examined the prognostic value of pretreatment platelet counts. It was consistently reported that high pretreatment platelet count was an independent prognostic factor for poor outcome (48–51). Platelets have been unarguably associated with cancer growth and metastasis (52). Platelet promotes angiogenesis, tumor growth and survival by interacting with tumor cells and tumor microenvironment (53). Activated platelets secrete proangiogenic proteins that promote endothelial growth and proliferation, hence promoting angiogenesis and tumor cell survival. Platelets also influence vascular tone and permeability, impacting the extravasation of tumor cells to distant regions and avoiding tumor hemorrhage. In shear stress, platelets secrete microparticles that promote cell adherence to the endothelium and tumor invasion. Tumor-derived proteins are preferentially sequestered and stored by platelets. Granulocyte-macrophage colony-stimulating factor (GM-CSF), tumor necrosis factor alpha (TNF-α), and transforming growth factor-beta 1(TGFβ1) promote tumor cell proliferation and survival. Platelets facilitate immune escapes by inhibiting the cytotoxicity of NK cells and interferon production by TGFβ1 and platelet-fibrin complex formation (53). Studies have reported that pretreatment PLR was associated with poorer OS (10, 54, 55). This is consistent with our results. We found that the higher the pretreatment PLR shorter the PFS. However, ΔmaxPLR during CCRT is associated with improved PFS (**Supplementary Figure 4**). This is likely driven by radiation-induced lymphopenia during CCRT as heatmap analysis has shown that ΔmaxPLR is negatively associated with lymphocyte counts (**Supplementary Figure 5**). All previous studies investigated the prognostic effects of pretreatment PLR. However, we evaluated the value of platelet changes during treatment for the first time and provided early evidence that dynamic changes in hematological markers may be of greater prognostic value than pretreatment values.

Interestingly, pretreatment EBV-DNA level was identified as a prognostic factor in univariate analysis. However, statistical significance was not achieved in subsequent multivariate analysis as it was found to be related to pretreatment GTVn volume in statistical analysis. Similar findings were reported by Jiang et al. (56). EBV-DNA levels provide information on tumor biological behavior beyond the anatomical staging. It has been hypothesized that the prognostic performance of the staging system can potentially be improved by including EBV-DNA levels (4). However, its wide application has been limited due to heterogeneity in laboratory techniques (57).

To our best knowledge, we are the first to include dynamic changes in hematological and inflammatory factors in constructing a nomogram. Our local protocol recommended induction chemotherapy with cisplatin 80mg/m^2^ every 3 weeks for 3 cycles and CCRT with cisplatin 100mg/m2 every 3 weeks for 2 cycles. Our nomogram identified patients who are at higher risk of progression. Adjuvant treatments, such as capecitabine or immunotherapy may be considered in those identified as high risk. This study has its limitations. All patients were treated in a single center in an endemic area for NPC. Results need to be validated externally and their application in non-endemic areas should be taken with caution. Second, measuring GTV is greatly impacted by the accuracy of diagnostic imaging, and the contouring of GTV may vary between operators. Computer algorithms and standardized imaging techniques may further improve GTV delineation accuracy in the future.

In conclusion, we developed and validated a nomogram for predicting PFS in patients with locally advanced NPC treated with induction chemotherapy and concurrent chemo-radiation. Our study is the first to validate dynamic hematological and inflammatory biomarkers as significant prognostic factors for NPC by comparing nomogram analyses. The proposed nomogram facilitates risk stratification and guides personalized treatment strategies, such as adjuvant treatment with capecitabine or immunotherapy.

## Data availability statement

The raw data supporting the conclusions of this article will be made available by the authors, without undue reservation.

## Ethics statement

The studies involving human participants were reviewed and approved by Clinical Research Ethics Committee of the University of Hong Kong -Shenzhen Hospital. The patients/participants provided their written informed consent to participate in this study.

## Author contributions

Conceptualization: QL, LY, and ZX. Methodology: QL, LM, HM, LY, and ZX. Data collection, analysis and interpretation: QL, LM, HM, LY, and ZX. Manuscript original draft and editing: QL. Manuscript review and editing: LM, LY, and ZX. Visualization: QL and LM. Supervision: LY and ZX. All authors contributed to the article and approved the submitted version.

## Funding

This project is supported in part by Shenzhen Science and Technology Program (No. ZDSYS20210623091811035 and JCYJ20210324114600002); Health Commission of Guangdong Province, China (NO. B2020100); High Level-Hospital Program, Health Commission of Guangdong Province, China (NO. HKUSZH201902031, HKUSZH201901017 and HKUSZH201901038) and Shenzhen Key Medical Discipline Construction Fund (No. SZXK014).

## Conflict of interest

The authors declare that the research was conducted in the absence of any commercial or financial relationships that could be construed as a potential conflict of interest.

## Publisher’s note

All claims expressed in this article are solely those of the authors and do not necessarily represent those of their affiliated organizations, or those of the publisher, the editors and the reviewers. Any product that may be evaluated in this article, or claim that may be made by its manufacturer, is not guaranteed or endorsed by the publisher.
