# Supplementary material for: Establishment of a prognostic nomogram for patients with locoregionally advanced nasopharyngeal carcinoma incorporating clinical characteristics and dynamic changes in hematological and inflammatory markers
Source: Front Oncol. 2022 Oct 26;12:1032213. doi: 10.3389/fonc.2022.1032213 (PMC9643849; doi:10.3389/fonc.2022.1032213)
Supplement: Supplementary file 1 [file DataSheet_1.pdf]

Supplementary Information for

Establishment and validation of a prognostic nomogram for patients with locoregionally advanced nasopharyngeal carcinoma incorporating clinical characteristics and dynamic changes in hematological and inflammatory markers during concurrent chemo-radiation

Qin Liu[1,2†], Lingyu Ma[1,2†], Huangrong Ma[1], Li Yang[1,2*] and Zhiyuan Xu[1,2*]

[1]Clinical Oncology Center, The University of Hong Kong-Shenzhen Hospital, Shenzhen, Guangdong Province, 518053, P.R.China.
[2]Shenzhen Key Laboratory of Translational Research on Recurrent/Metastatic Cancer, The University of Hong Kong - Shenzhen Hospital. Shenzhen, Guangdong Province, 518053, P.R.China.

[*]Correspondence:
Yang Li, PhD
yangl1@hku-szh.org
Zhiyuan Xu, MD
xuzy@hku-szh.org

[†]These authors contributed equally to this work

Table of Contents

Supplementary Tables 1-8

Supplementary Figures 1-5

Δp/bLYM	Lymphocyte count post-IC/baseline lymphocyte count
Δe/bLYM	Lymphocyte count at end of treatment/baseline lymphocyte count
Δe/pLYM	Lymphocyte count at end of treatment/lymphocyte post-IC
ΔminLYM	Minimum lymphocyte count during CCRT/lymphocyte count post-IC
Δp/bPLT	Platelet count post-IC/baseline platelet count
Δe/bPLT	Platelet count at end of treatment/baseline platelet count
Δe/pPLT	Platelet count at end of treatment/platelet post-IC
ΔminPLT	Minimum platelet count during CCRT/ platelet count post-IC
Δp/bNEUT	Neutrophil count post-IC/baseline neutrophil count
Δe/bNEUT	Neutrophil count at end of treatment/baseline neutrophil count
Δe/pNEUT	Neutrophil count at end of treatment/neutrophil post-IC
ΔminNEUT	Minimum neutrophil count during CCRT/neutrophil count post-IC
Δp/bHGB	Hemoglobin count post-IC/baseline hemoglobin count
Δe/bHGB	Hemoglobin count at end of treatment/baseline hemoglobin count
Δe/pHGB	Hemoglobin count at end of treatment/hemoglobin post-IC
ΔminHGB	Minimum hemoglobin count during CCRT/hemoglobin count post-IC
Δp/bNLR	NLR post-IC/baseline NLR<br>NLR (neutrophil-to-lymphocyte ratio) = neutrophil count (10^9/L)/lymphocyte count (10^9/L)
Δe/bNLR	NLR at end of treatment/baseline NLR
Δe/pNLR	NLR at end of treatment/NLR post-IC
ΔmaxNLR	Maximum NLR during CCRT/NLR post-IC
Δp/bSII	SII post-IC/baseline SII<br>SII (systemic inflammatory index) = platelet count (10^9/L) x neutrophil count (10^9/L)/ lymphocyte count (10^9/L)
Δe/bSII	SII at end of treatment/baseline SII
Δe/pSII	SII at end of treatment/SII post-IC
ΔmaxSII	Maximum SII during CCRT/SII post-IC
Δp/bPLR	PLR post-IC/baseline PLR<br>PLR (platelet-to-lymphocyte ratio) = platelet count (10^9/L)/lymphocyte count (10^9/L)
Δe/bPLR	PLR at end of treatment/baseline PLR
Δe/pPLR	PLR at end of treatment/PLR post-IC
ΔmaxPLR	Maximum PLR during CCRT/PLR post-IC
Δp/bCA	Calcium level post-IC/baseline calcium level

Supplementary Table 1. Calculations of dynamic changes in hematological and inflammatory markers

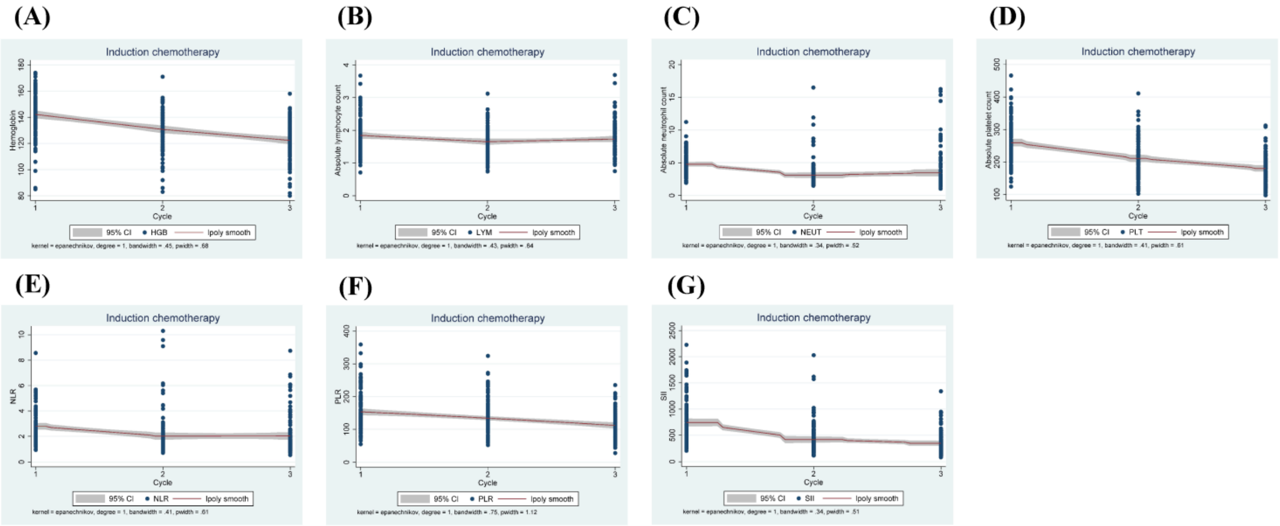

Supplementary Figure 1. Changes in hematological and inflammatory markers during induction chemotherapy, including hemoglobin(A), lymphocyte(B), neutrophil(C), platelet(D), NLR(E), PLR(F) and SII(G).

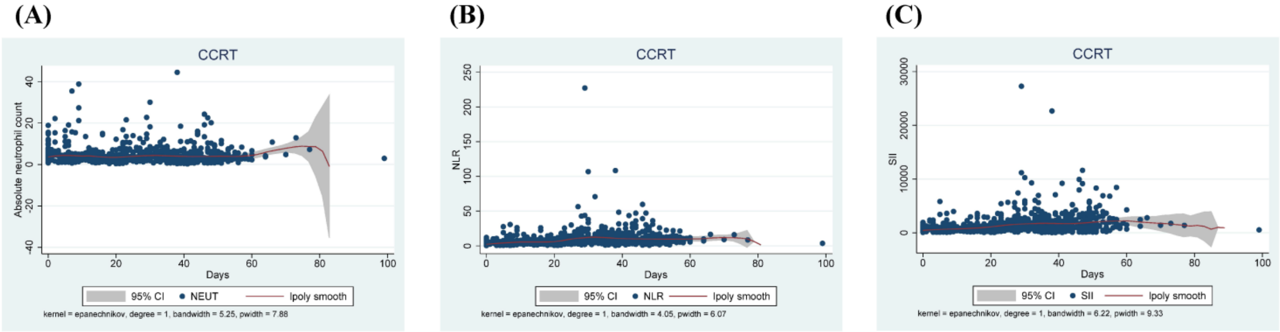

Supplementary Figure 2. Changes in neutrophils(A), NLR(B), SII(C) during concurrent chemo-radiation.

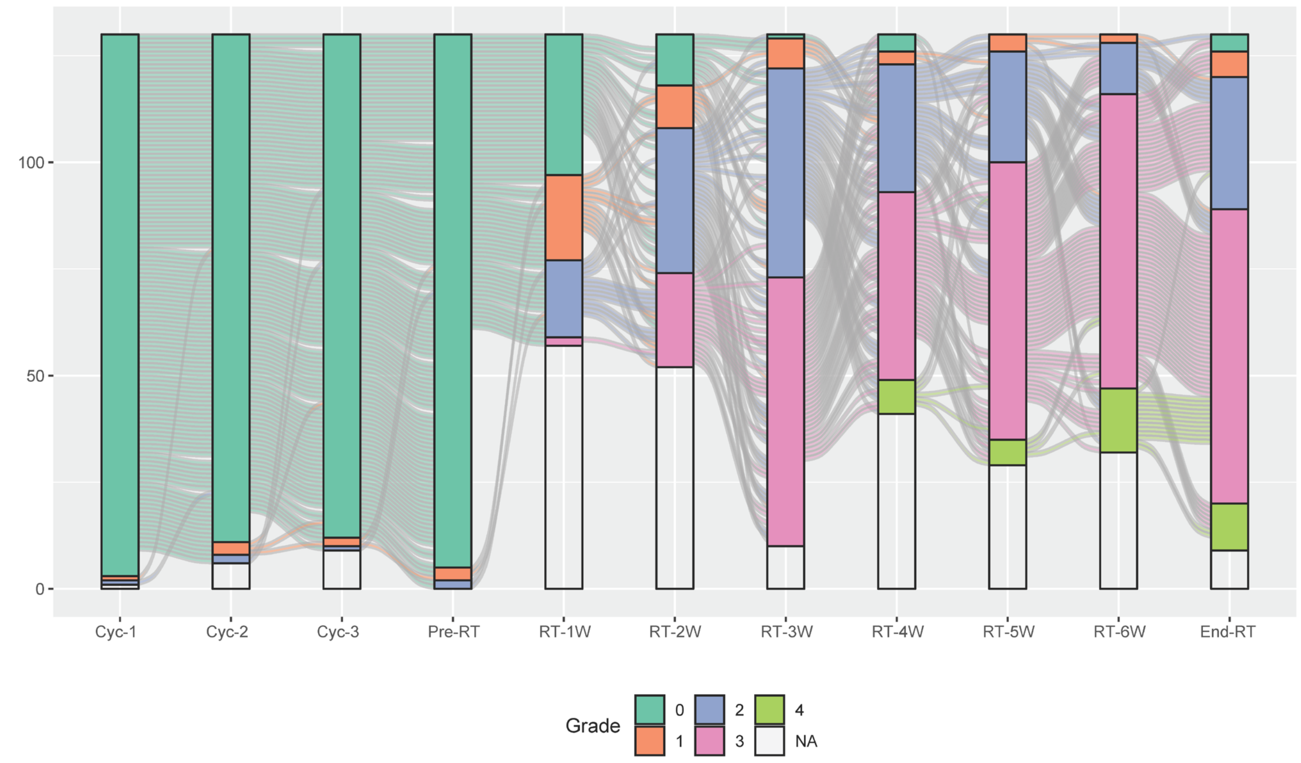

Supplementary Figure 3. Changes in degree of lymphopenia during treatment. Lymphopenia was graded according to the CTCAE v4.0.

Induction chemotherapy	Coefficient (95%CI)	*P*-value
Continue	-0.054 (-0.097, -0.012)	0.012
Baseline	Reference	
Cycle 2	-0.178 (-0.255, -0.102)	<0.001
Cycle 3	-0.106 (-0.191, -0.021)	0.015
Concurrent chemo-RT	Coefficient (95%CI)	
Continue	-0.163 (-0.175, -0.152)	<0.001
Pre-RT	Reference	
Week 1	-0.623 (-0.731, -0.515)	<0.001
Week 2	-0.997 (-1.091, -0.903)	<0.001
Week 3	-1.169 (-1.262, -1.076)	<0.001
Week 4	-1.208 (-1.308, -1.074)	<0.001
Week 5	-1.259 (-1.352, -1.167)	<0.001
Week 6	-1.347 (-1.442, -1.252)	<0.001
End-RT	-1.226 (-1.327, -1.126)	<0.001

Supplementary Table 2. Kinetics of absolute lymphocyte counts during induction chemotherapy and concurrent chemo-radiation

Induction chemotherapy	Coefficient (95%CI)	*P*-value
Continue	-40.212 (-44.993, -35.430)	<0.001
Pre-Chemo	Reference	
Cycle 2	-49.467 (-57.259, -41.675)	<0.001
Cycle 3	-80.199 (-89.791, -70.608)	<0.001
Concurrent chemo-RT	Coefficient (95%CI)	
Continue	1.296 (1.100, 1.493)	<0.001
Pre-RT		
Week 1	1.917 (0.921, 2.914)	<0.001
Week 2	3.771 (3.028, 4.514)	<0.001
Week 3	2.633 (2.097, 3.169)	<0.001
Week 4	9.976 (4.973, 14.979)	<0.001
Week 5	10.559 (8.243, 12.875)	<0.001
Week 6	7.696 (6.179, 9.213)	<0.001
End-RT	7.556 (6.320, 8.792)	<0.001

Supplementary Table 3. Kinetics of absolute platelet counts during induction chemotherapy and concurrent chemo-radiation

Induction chemotherapy	Coefficient (95%CI)	*P*-value
Continue	-0.661 (-0.971, -0.352)	<0.001
Pre-Chemo	Reference	
Cycle 2	-1.667 (-2.133, -1.202)	<0.001
Cycle 3	-1.303 (-1.924, -0.682)	<0.001
Concurrent chemo-RT	Coefficient (95%CI)	
Continue	0.035 (-0.038, 0.108)	0.348
Pre-RT	Reference	
Week 1	0.287 (-0.673, 1.246)	0.558
Week 2	0.149 (-0.542, 0.839)	0.673
Week 3	-1.116 (-1.716, -0.516)	<0.001
Week 4	0.450 (-0.194, 1.094)	0.171
Week 5	1.085 (0.450, 1.721)	0.001
Week 6	-0.563 (-1.234, 0.108)	0.100
End-RT	0.273 (-0.410, 0.957)	0.433

Supplementary Table 4. Kinetics of absolute neutrophil counts during induction chemotherapy and concurrent chemo-radiation

Induction chemotherapy	Coefficient (95%CI)	*P*-value
Continue	-9.875 (-10.994, -8.757)	<0.001
Pre-Chemo	Reference	
Cycle 2	-11.161 (-12.761, -9.562)	<0.001
Cycle 3	-19.718 (-21.955, -17.481)	<0.001
Concurrent chemo-RT	Coefficient (95%CI)	
Continue	-2.509 (-2.901, -2.116)	<0.001
Pre-RT	Reference	
Week 1	1.102 (-0.940, 3.145)	0.290
Week 2	-9.643 (-11.484, -7.803)	<0.001
Week 3	-11.342 (-13.015, -9.669)	<0.001
Week 4	-8.369 (-10.408, -6.329)	<0.001
Week 5	-12.814 (-15.315, -10.312)	<0.001
Week 6	-20.383 (-23.106, -17.660)	<0.001
End-RT	-14.866 (-17.580, -12.151)	<0.001

Supplementary Table 5. Kinetics of hemoglobin during induction chemotherapy and concurrent chemo-radiation

Induction chemotherapy	Coefficient (95%CI)	*P*-value
Continue	-0.383 (-0.549, -0.217)	<0.001
Pre-Chemo	Reference	
Cycle 2	-0.770 (-1.090, -0.451)	<0.001
Cycle 3	-0.758 (-1.090, -0.426)	<0.001
Concurrent chemo-RT	Coefficient (95%CI)	
Continue	2.481 (0.730, 4.233)	0.005
Pre-RT	Reference	
Week 1	-11.094 (-20.869, -1.319)	0.026
Week 2	-44.208 (-55.406, -33.011)	<0.001
Week 3	-19.728 (-28.546, -10.909)	<0.001
Week 4	10.690 (-3.776, 25.156)	0.148
Week 5	-12.381 (-27.062, 2.300)	0.098
Week 6	-38.967 (-52.324, -25.610)	<0.001
End-RT	33.048 (18.524, 47.573)	<0.001

Supplementary Table 6. Kinetics of NLR during induction chemotherapy and concurrent chemo-radiation

Induction chemotherapy	Coefficient (95%CI)	*P*-value
Continue	-200.594 (-239.420, -161.768)	<0.001
Pre-Chemo	Reference	
Cycle 2	-329.570 (-405.963, -253.176)	<0.001
Cycle 3	-398.507 (-475.864, -321.150)	<0.001
Concurrent chemo-RT	Coefficient (95%CI)	
Continue	246.008 (210.125, 281.890)	<0.001
Pre-RT	Reference	
Week 1	309.829 (164.839, 454.819)	<0.001
Week 2	430.763 (313.576, 547.951)	<0.001
Week 3	402.904 (307.987, 497.822)	<0.001
Week 4	1693.160 (1059.006, 2327.315)	<0.001
Week 5	1691.175 (1367.258, 2015.092)	<0.001
Week 6	1026.197 (753.922, 1298.473)	<0.001
End-RT	1701.652 (1398.537, 2004.766)	<0.001

Supplementary Table 7. Kinetics of SII during induction chemotherapy and concurrent chemo-radiation

Induction chemotherapy	Coefficient (95%CI)	*P*-value
Continue	-21.023 (-25.554, -16.493)	<0.001
Pre-Chemo	Reference	
Cycle 2	-19.036 (-27.597, -10.474)	<0.001
Cycle 3	-42.095 (-51.167, -33.024)	<0.001
Concurrent chemo-RT	Coefficient (95%CI)	
Continue	67.296 (60.697, 73.894)	<0.001
Pre-RT	Reference	
Week 1	56.599 (39.309, 73.890)	<0.001
Week 2	106.015 (83.153, 128.877)	<0.001
Week 3	230.986 (204.159, 257.813)	<0.001
Week 4	373.914 (312.909, 434.919)	<0.001
Week 5	342.554 (291.138, 393.970)	<0.001
Week 6	385.379 (330.940, 439.818)	<0.001
End-RT	460.227 (399.086, 521.367)	<0.001

Supplementary Table 8. Kinetics of PLR during induction chemotherapy and concurrent chemo-radiation

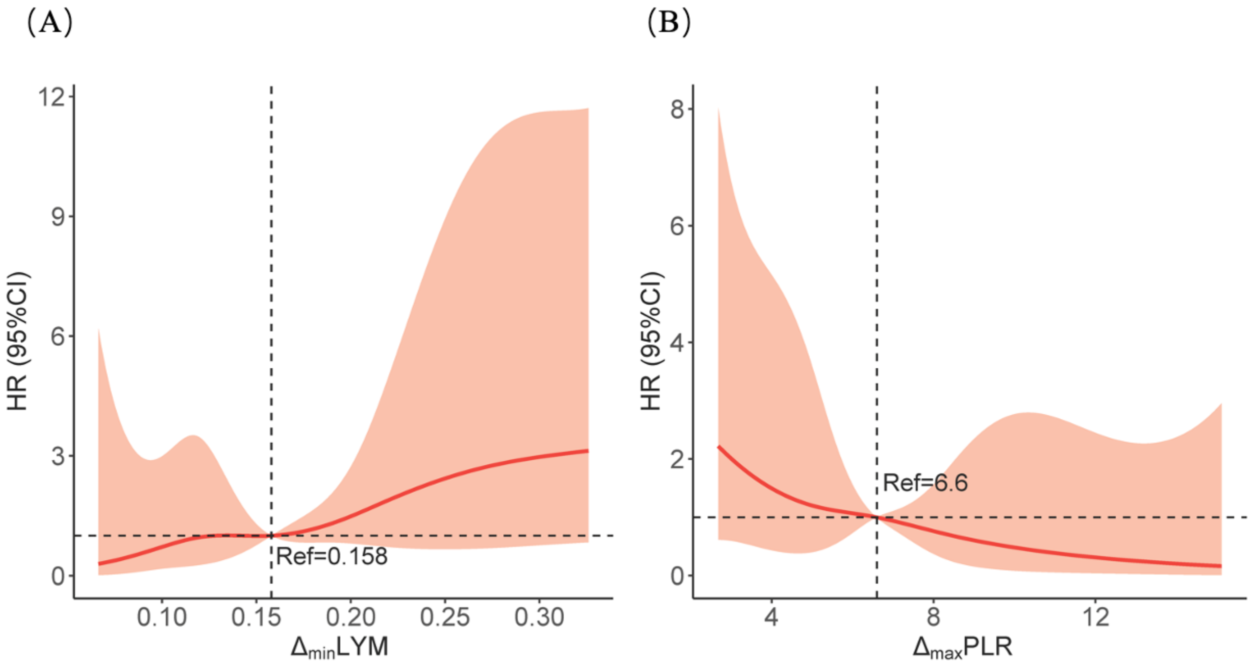

Supplementary Figure 4. Non-linear relationships between ΔminLYM and PFS (A) and between ΔmaxPLR and PFS (B) were modelled using RCS.

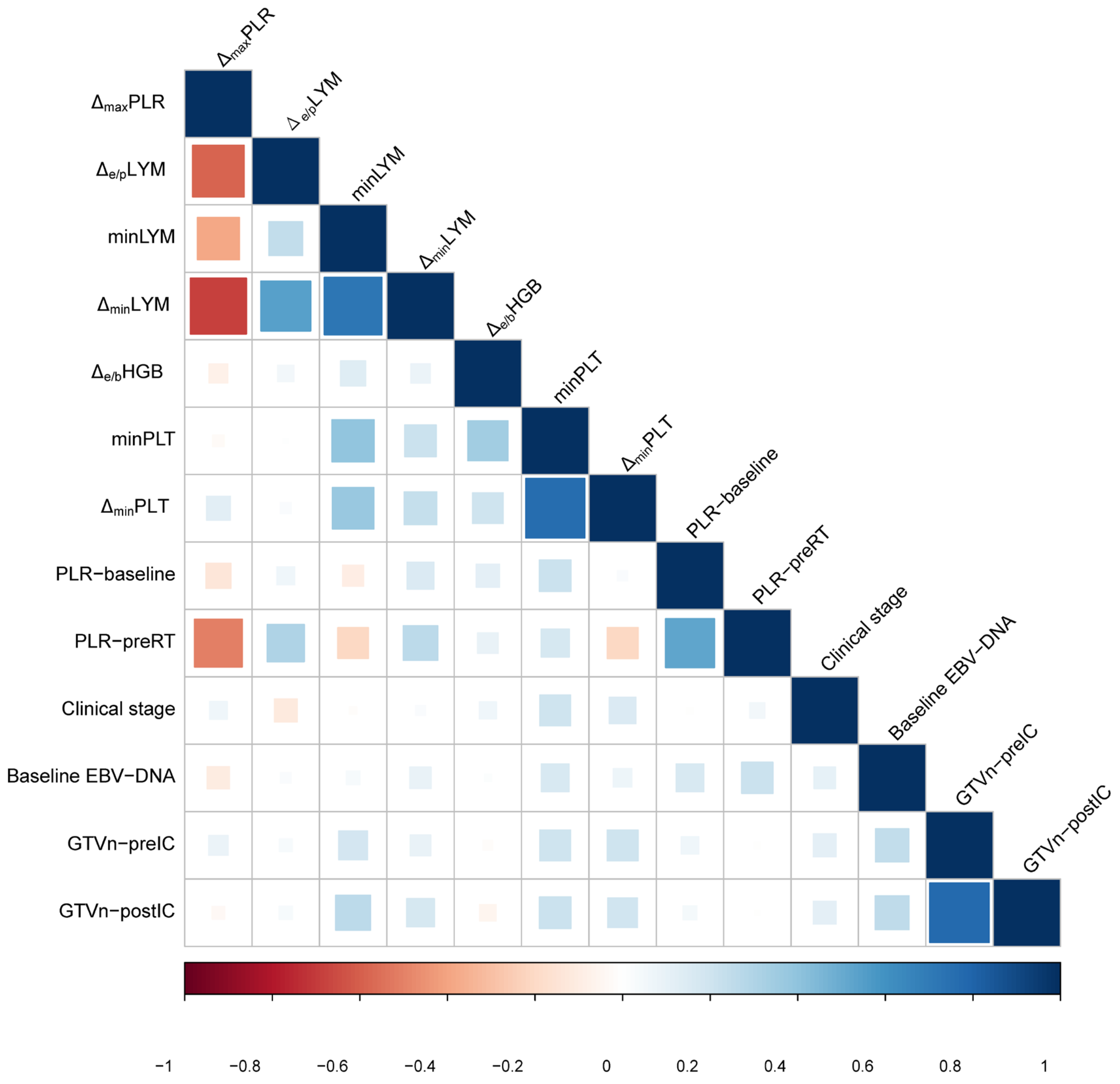

Supplementary Figure 5. Covariate-adjusted heatmap analysis
